# Self-perceived fatigue in relation to activity and participation in adolescents and adults with cerebral palsy living in urban South Africa

**DOI:** 10.1177/18758894251406431

**Published:** 2025-12-23

**Authors:** Nelleke Gertrude Langerak, Roshaan Salie, Kirsten Ann Donald, Anthony Graham Fieggen, Maaike Maria Eken

**Affiliations:** 1 Neuroscience Institute, Faculty of Health Sciences, 6034 University of Cape Town, Cape Town, South Africa; 2 Division of Neurosurgery, Faculty of Health Sciences, 37716 University of Cape Town, Cape Town, South Africa; 3 Department of Research, Sint Maartenskliniek, Nijmegen, The Netherlands; 4 Department of Medical BioSciences, Physiology, Radboud university medical center, Nijmegen, The Netherlands; 5 Division of Developmental Paediatrics, Department of Paediatrics and Child Health, Faculty of Health Sciences, 37716 University of Cape Town, Cape Town, South Africa; 6 Division of Sport and Excercise Medicine, Department of Exercise, Sport and Lifestyle Medicine, Faculty of Medicine and Health Sciences, 121470 Stellenbosch University, Cape Town, South Africa

**Keywords:** cerebral palsy, adolescents, adults, fatigue, participation

## Abstract

**Purpose:**

Understanding the level of fatigue experienced by adolescents and adults with cerebral palsy (CP) in low- and middle-income countries is crucial, as it can inform healthcare workers in the assessment, prevention, and management of fatigue for these individuals, similar to the approach taken in high-income countries. This study aimed to determine self-perceived fatigue and the level of accomplishments and satisfaction of activities and participation in daily life in adolescents and adults with CP compared with typically developing (TD) peers living in urban South Africa. The study also examined whether the outcome measures were associated within the CP cohort.

**Methods:**

This case-control study included 31 adolescents and 30 adults with CP and TD peers matched for age, sex, and socio-economic status. Participants completed the Fatigue Severity Scale and Life-Habits Questionnaire.

**Results:**

Self-perceived fatigue was reported in 14/31 adolescents with CP, 6/31 TD adolescents, 9/30 adults with CP, and 8/30 TD peers. No differences in fatigue were observed between adults or adolescents with CP and TD peers. However, accomplishment and satisfaction scores were lower for adolescents (p < 0.001 and p = 0.016, respectively) and adults with CP (both p < 0.001) compared to TD peers.

**Conclusions:**

Individuals with CP living in urban South Africa reported similar levels of fatigue as TD peers. Despite limitations in accomplishing life habits, adolescents and adults with CP reported to be independent in their daily activities and satisfied with their community participation, which was unrelated to fatigue.

## Introduction

Although cerebral palsy (CP) is considered one of the most common childhood disabilities worldwide, and traditionally associated with greater morbidity and mortality,^1^ recent literature suggests that individuals with CP now have a life expectancy comparable to typically developing (TD) peers.^2,3^ However, with improved life expectancy, adults with CP may develop health complications as they age, often arising from the long-term consequences of CP. Previous literature reports a gradual decline in perceived health and functional level in adults with CP,^3^ with fatigue reported as one of the most common health issues in adults with CP.^4^

Self-perceived fatigue, a measurement of one's own perception of fatigue and how it affects one's performance in daily life, is higher in individuals with CP compared to their TD peers.^5^ In addition, fatigue reported by individuals with CP is associated with pain, CP-related factors (spasticity, gross motor function level), sleep, mental health concerns,^6^ and low levels of satisfaction.^7^ These studies on self-perceived fatigue in adults with CP are, however, most commonly conducted in high-income countries (HICs), while little literature is available from low- to middle-income countries (LMICs). It is uncertain whether the same findings of HICs apply in LMICs, as experiences may be shaped by contextual factors such as healthcare, daily environmental demands, and societal expectations.

In a series of studies conducted with the same cohort of adolescents and adults with CP across Gross Motor Function Classification System (GMFCS) levels I – V residing in urban South Africa, it was found that these individuals reported similar physical activity levels^8^ and mental health^9^ compared to their TD peers. Furthermore, when the adult cohort was interviewed regarding their unmet needs in daily living, fatigue did not emerge as one of the most important challenges faced.^10^ Although fatigue did not appear as one of the most important challenges, the level of fatigue that adolescents and adults with CP who live in LMICs experience is still unknown. As previously reported in HICs, it is crucial to understand the level of fatigue that these individuals experience and to what extent it may affect their lives. This may guide healthcare workers for examination, prevention, and management of the individual.

Therefore, this study aimed to determine self-perceived fatigue of adolescents and adults living with CP compared to TD peers in urban South Africa. A second aim was to determine and compare the levels of accomplishment and satisfaction of daily activities and participation in these cohorts. Finally, the study aimed to assess whether there was an association between self-perceived fatigue and level of accomplishment and satisfaction in individuals with CP. It was hypothesized that adolescents and adults with CP living in an urban South African context would report higher levels of self-perceived fatigue compared to their TD peers, and greater fatigue would be associated with lower accomplishment and satisfaction in daily activities and participation. However, given contextual differences such as healthcare access, environmental demands, and societal expectations, these patterns may differ from those reported in HICs.

## Methods

### Study design and participants

This case-control study was a sub-study of a bigger research project including adolescents and adults with CP and matched TD peers living in Cape Town, South Africa, focusing on their challenges, needs, and well-being.^8,9,10^ With regard to the adolescent cohort, a convenience sample of adolescents with CP was recruited from three schools that educate Learners with Special Education Needs (LSEN) and that follow a mainstream curriculum. Adolescents had to be enrolled in their last three years of high school. TD adolescents were matched for age, gender, and socio-economic status (SES), and were recruited from mainstream secondary schools in Cape Town.

The sample of adults with CP was identified through databases of former research projects,^11,12^ direct referrals, word-of-mouth recommendations, and social media strategies. These adults were included if they were 23–40 years old and had attended a LSEN school at least five years prior to the study. Similar methods were used to recruit TD adults who were matched for age, sex, and SES prior to enrollment. Participants needed to be able to communicate in either English or Afrikaans, and to live within 100 kilometers of Cape Town. Exclusion criteria for both individuals with CP and TD peers included a diagnosis of a movement disorder other than CP, peripheral neuromuscular disorder, or other disorder resulting in physical impairments.

All individuals who fulfilled the inclusion criteria and expressed a willingness to participate in the study provided written informed consent. The University of Cape Town Human Research Ethics Committee granted approval for the study (HREC 014/2017), and it was conducted in accordance with the Declaration of Helsinki's guiding principles.^13^

### Assessments

Participants’ background information was obtained, including age, sex, body mass index, subtype of CP, and SES. SES was estimated using a housing index, which was calculated by dividing the number of people living in the house by the number of rooms in the house, excluding the kitchen and bathroom.^10^ The housing index was categorized as follows: < 1.0: “high SES”; between 1.0 and 1.5: “average SES”; and >1.5: “low SES”.^14^ This index has been successfully used in studies conducted in urban South Africa.^9,10,15^ In addition, information regarding participants’ subtype of CP, GMFCS level,^16^ Manual Ability Classification System (MACS) level,^17^ and Communication Function Classification System (CFCS) level^18^ were collected.

To describe the level of mobility in participants’ daily environment, the Functional Mobility Scale (FMS) was used. Participants were assigned a score for three distances, including five, 50, and 500 meters (m). Scores range from 1 to 6, with level 1 indicating use of a wheelchair; level 2 use of a walker or frame; level 3 use of crutch(es); level 4 use of stick(s) or furniture, walls, fences, or shopfronts for support; level 5 independent on level surfaces; and level 6 independent on all surfaces.^19^ It has been demonstrated that the FMS is a valid and reliable measure to estimate functional mobility in individuals with CP.^19^

### Fatigue severity scale (FSS)

Self-perceived fatigue was identified with the FSS. This is a self-administered questionnaire in which participants are asked to rate nine items on a seven-point Likert scale. The scores range from 1, indicating strongly disagree, to 7, indicating strongly agree. Severe fatigue was defined as a mean FSS score ≥5.1 and fatigue as ≥4.0, as has been used in previous studies with adolescents^20,21^ as well as adults with CP.^22,23^ The FSS's internal consistency, reliability, validity, and sensitivity have been demonstrated in different patient populations.^24^

### Life-habits questionnaire 3.1 (Life-H)

The level of accomplishment and satisfaction of daily activities and participation among the individuals was assessed with the Life-H.^25^ This questionnaire asks participants to rate their accomplishment and satisfaction on 77 life habits related to 12 dimensions: Nutrition, Fitness, Personal Care, Communication, Housing, Mobility, Responsibilities, Interpersonal Relationships, Community Life, Education, Employment and Recreation.

Accomplishment scores range from 0 (not accomplished or achieved) to 9 (accomplished without difficulty and without assistance). Satisfaction scores range from −10 (very unsatisfied) to 10 (very satisfied).^25^ For each dimension, a weighted score (taking into account situations when a particular life habit was not applicable) per dimension, as well as a total weighted score for Accomplishment and Satisfaction, were derived.^25^ The Life-H questionnaire has been shown to have good validity and reliability,^25^ and has been used in numerous cohort studies of individuals with CP, including in LMICs.^12,15^

### Statistical analysis

Participants’ demographics were reported using descriptive statistics. Based on the Shapiro-Wilk test results, non-parametric statistical analyses (median [interquartile ranges (IQRs)]) were utilized to describe the participants’ socio-demographic data. Mann-Whitney U tests were performed to determine a difference in the levels of fatigue (FSS mean score) between individuals with CP and TD peers in the adolescent and adult cohorts. Similar statistical analyses were completed to determine differences in levels of activity and participation for accomplishment and satisfaction levels (Accomplishment and Satisfaction Life-H total weighted scores).

Spearman rho correlations were calculated to determine associations between self-perceived fatigue (FSS mean score) of adolescents and adults with CP and their levels of activity and participation (Accomplishment and Satisfaction Life-H total weighted scores). A Bonferroni correction was applied to correct for multiple analyses, resulting in the significance set at p < 0.025 (0.05/2). Statistical analyses were conducted using SPSS (version 25; IBM SPSS Inc, Chicago, IL, USA).

## Results

### Background information

Participant characteristics are presented in Table 1, exhibiting the purposeful matching for age, sex and SES of the CP and TD cohorts. The adolescents with CP (n = 31) were classified across GMFCS levels I/II/III/IV/V: 6/9/6/5/5; MACS: 21/5/2/2/1; and CFCS: 22/8/0/1/0. The adult CP cohort (n = 30) included participants classified with levels I/II/III/IV/V for GMFCS: 6/6/5/7/6; MACS: 23/4/2/1/0; and CFCS: 24/4/1/1/0.

**Table 1.** Overview of participant characteristics.

Parameters	Adolescents		Adults	
	CP (n = 31)	TD (n = 31)	CP (n = 30)	TD (n = 30)
**Age**, median [IQR]	17y9m [16y6m– 18y6m]	17y3m [16y11m– 18y2m]	34y10m [30y2 m −36y1m]	33y11m [29y3m–35y6m]
**Sex**, n (%)				
Male	15 (48)	15 (48)	10 (33)	10 (33)
Female	16 (52)	16 (52)	20 (67)	20 (67)
**SES**				
Median [IQR]	1.3 [1.0–1.8]	1.3 [0.9–1.5]	1.3[ 1.0–1.7]	1.3 [1.0–1.7]
Categories, n (%)				
Low	9 (29)	6 (19)	8 (27)	9 (30)
Average	18 (58)	17 (55)	17 (57)	18 (60)
High	4 (13)	8 (26)	5 (17)	3 (10)
**BMI**				
Median [IQR]	19.8 [18.0–23.5]	21.2 [19.0–25.6]	28.5[23.5–35.8]	28.0 [22.9–32.9]
Categories, n (%)				
Underweight	10 (32)	5 (16)	1 (3)	1 (3)
Healthy	14 (45)	16 (52)	11 (37)	9 (30)
Overweight	4 (13)	8 (26)	6 (20)	11 (37)
Obese	3 (10)	2 (7)	12 (40)	9 (30)
**Subtype of CP**, n (%)				
Unilateral spastic CP	10 (32)	n/a	2 (7)	n/a
Bilateral spastic CP	16 (52)	n/a	25 (83)	n/a
Dyskinesia	5 (16)	n/a	3 (10)	n/a

CP: cerebral palsy; TD: typically developing; IQR: interquartile range; SES: socio-economic status; BMI: body mass index; and n/a: not applicable.

With regard to the FMS, almost half of the adolescents with CP (n = 15; 48%) were able to walk independently (FMS score 5 or 6) for 5 m, 50 m, and 500 m, while a third used a wheelchair (FMS score 1) for long distances. Regarding the adults with CP, slightly fewer were able to walk independently over 5 m (40%), 50 m and 500 m (37%), and slightly more adults used a wheelchair for long distances (47%) (Table 2).

**Table 2.** Overview of functional mobility scale (FMS) scores for adolescents and adults with cerebral palsy.

FMS	Adolescents (n = 31)			Adults (n = 30)		
	5 meter	50 meter	500 meter	5 meter	50 meter	500 meter
1	6 (19)	9 (29)	11 (35)	6 (20)	11 (37)	14 (47)
2	4 (13)	2 (6)	1 (3)	1 (3)	4 (13)	3 (10)
3	5 (16)	5 (16)	4 (13)	1 (3)	1 (3)	1 (3)
4	1 (3)	0 (0)	0 (0)	10 (33)	3 (10)	1 (3)
5	1 (3)	8 (26)	10 (32)	2 (7)	3 (10)	7 (23)
6	14 (45)	7 (23)	5 (16)	10 (33)	8 (27)	4 (13)

Score 1: Use of a wheelchair; 2: Use of a walker or frame; 3: Use of crutches; 4: Use of (1 or 2) sticks; 5: Independent on level surfaces; and 6: Independent on all surfaces.

### Self-perceived fatigue

**Figure 1.**
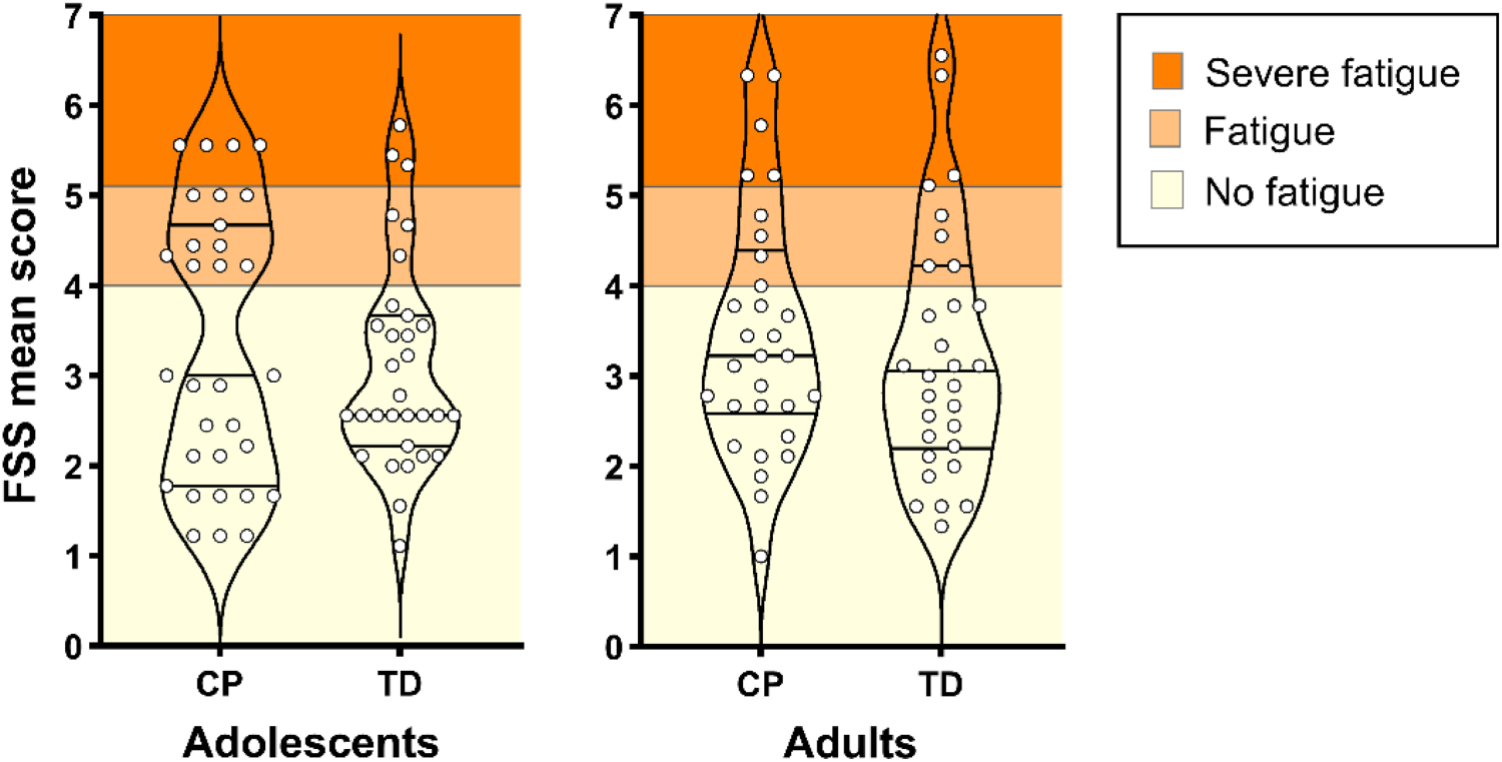
Levels of fatigue among adolescent and adult participants as measured by the FSS. Violin plots display the distribution of scores, with horizontal lines indicating the median and interquartile ranges. Abbreviations: FSS, Fatigue Severity Scale; CP, cerebral palsy; and TD, typically developing.

Self-perceived fatigue was reported in 14 adolescents with CP (13% severe fatigue, 32% fatigue), six TD adolescents (10% severe fatigue and 10% fatigue), nine adults with CP (17% severe fatigue and 13% fatigue) and eight TD peers (13% severe fatigue and 13% fatigue). Table 3 provides an overview of the median [IQR] of the FSS mean scores for each cohort, showing no differences between the levels of fatigue of the adolescents and adults with CP compared to TD peers (Figure 1).

**Table 3.** Differences in FSS and Life-H total weighted scores between adolescents and adults with CP and TD peers.

	Adolescents			Adults		
	CP (n = 31)	TD (n = 31)		CP (n = 30)	TD (n = 30)	
	Median [IQR]	Median [IQR]	*p*	Median [IQR]	Median [IQR]	*p*
FSS						
FSS Mean Scores	3.0 [1.8–4.7]	2.6 [2.2–3.7]	0.800	3.2 [2.6–4.4]	3.1 [2.2–4.2]	0.473
Life H						
Level of Accomplishment	8.3 [6.0–9.4]	9.5 [9.4–9.6]	***<0*** **.** 001*	8.4 [7.0–9.2]	9.9 [9.6–10.0]	***<0*** **.** 001*
Level of Satisfaction	6.5 [5.2–8.8]	9.0 [6.1–9.2]	***0*** **.** 016*	5.6 [5.0–7.8]	9.1 [6.6–9.7]	***<0*** **.** 001*

FSS: Fatigue Severity Scale; Life-H: Life-Habits Questionnaire; CP: cerebral palsy; TD: typically developing; and IQR: interquartile range. *Significant p *<* *0.025.*

### Life habits

**Figure 2.**
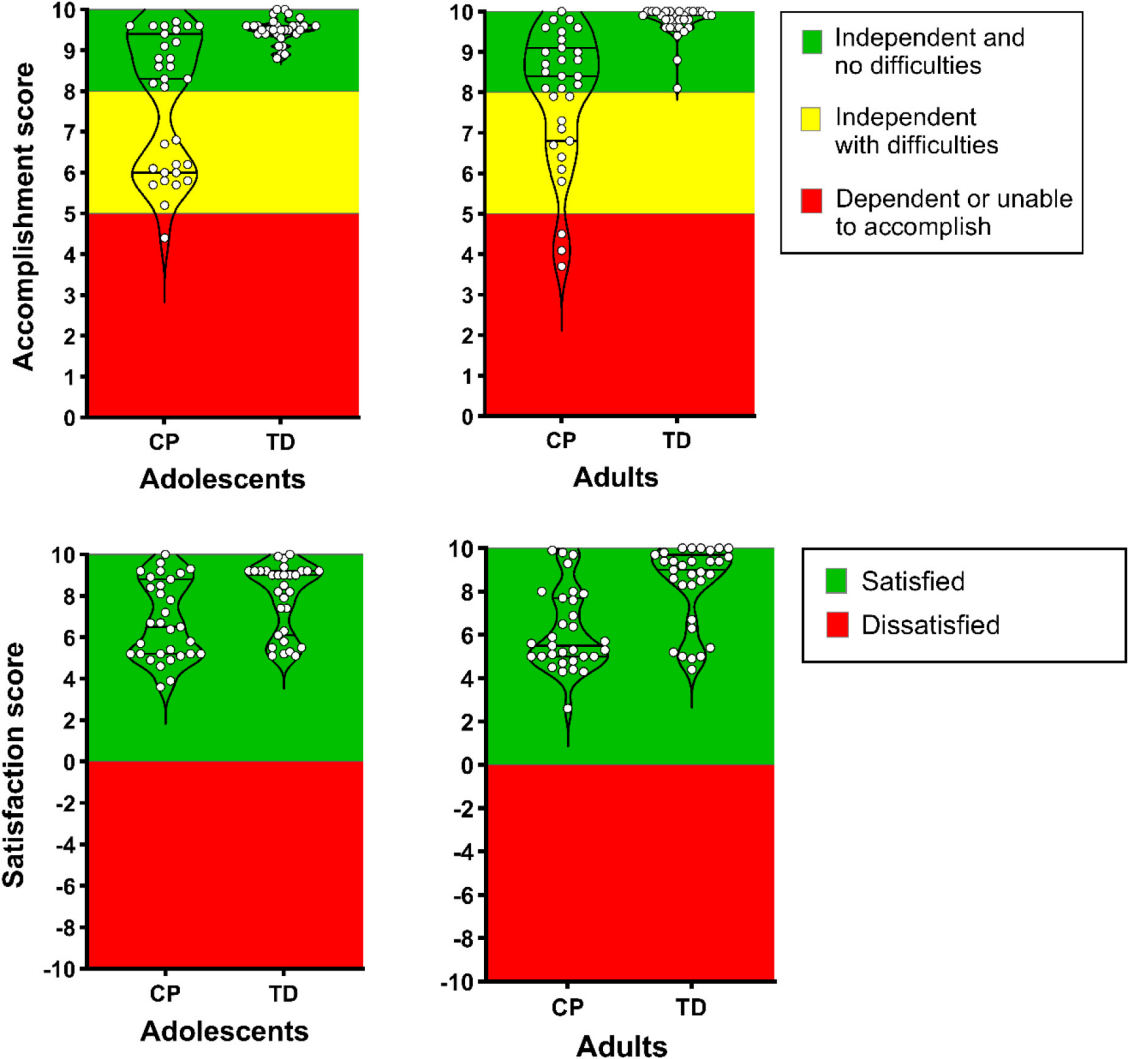
Levels of accomplishment and satisfaction among adolescent and adult participants as measured by the weighted scores of the Life-Habits Questionnaire. Violin plots display the distribution of scores, with horizontal lines indicating the median and interquartile ranges. Abbreviations: CP, cerebral palsy; and TD, typically developing.

All but one adolescent with CP reported accomplishing all life habits independently, with just over half reporting no difficulties (n = 18; 58%). Also, a great proportion of adults with CP (n = 27; 90%) reported accomplishing all life habits independently, with most of them reporting no difficulties (n = 19; 63%). As expected, all TD adults and adolescents reported being independent. With regards to the level of satisfaction, all participants indicated that they were generally satisfied with the way they accomplished the life habits explored in this study (Figure 2).

Detailed information per dimension for the four cohorts is provided in Appendices A and B. As presented in Table 3, total weighted accomplishment and satisfaction scores were significantly lower for both adolescents and adults with CP compared to TD peers.

### Associations

No associations were observed between self-perceived fatigue and levels of accomplishment nor satisfaction for adolescents or adults with CP (Table 4).

**Table 4.** Spearman correlations for total weighted scores of adolescents and adults with CP **FSS** versus **Life-H** level of accomplishment and level of satisfaction.

		Adolescents with CP (n = 31)	Adults with CP (n = 30)
Level of		FSS Mean Scores	FSS Mean Scores
Accomplishment	rho	0.178	−0.286
	*p*	0.337	0.126
Satisfaction	rho	−0.006	−0.300
	*p*	0.969	0.107

CP: cerebral palsy; FSS: Fatigue Severity Scale; and Life-H: Life-Habits Questionnaire.

## Discussion

This study is one of the first to describe similar levels of fatigue reported by adolescents and adults with CP (GMFCS level I – V) compared to TD peers living in a resource-limited environment. Both adolescents and adults with CP who participated in this study reported lower levels of accomplishment with their life habits compared to their TD peers. However, overall, the majority reported no challenges in accomplishing daily tasks or in their participation in the community and were satisfied with how they accomplished them. These accomplishment and satisfaction levels of the adolescents and adults with CP were not associated with self-perceived fatigue.

### Self-perceived fatigue

Self-perceived fatigue reported by adolescents with CP in the current study was similar to TD peers. This contrasted with findings in the literature based on studies conducted in HICs, where individuals with CP reported more fatigue than their peers.^5^ The current study showed that 30% of adults with CP reported self-perceived fatigue, while in previous studies conducted in a HIC such as the Netherlands, this percentage ranged from 50%^23^ to 61%.^26^ In addition, the severity of self-perceived fatigue also seemed lower in the current study (FSS 3.2) than in studies conducted in HICs (FSS 3.8–4.4).^22,23,27^

Interestingly, fatigue was not mentioned as one of the key issues in a qualitative study on the needs of this same cohort of adults with CP living in urban South Africa,^10^ while it has been recognized as one of the most limiting factors of adults with CP living in HICs.^4^ In addition, another recent study on the same cohort found that adults with CP reported good mental health (SF-36) and did not show more mental health symptoms (depression, anxiety and self-efficacy) than their TD peers,^9^ in contrast to what has been described in the literature.^6^ These differences could be related to cultural diversity and differences in coping mechanisms between individuals with CP living in LMICs and HICs, and therefore, perspectives could be shaped by circumstances. More research is needed to draw firm conclusions about differences in fatigue reported by individuals with CP living in HICs and LMICs.

### Activity and participation

The results of this study showed that the activity and participation of adolescents and adults with CP were relatively good. More specifically, individual scores showed that adolescents and adults with CP mostly reported accomplishing life habits independently without any difficulties and were relatively satisfied with the accomplishment of their life habits. Although individual scores were good, as expected, both adolescents and adults with CP reported lower total weighted scores for accomplishments and satisfaction compared to TD peers. This is consistent with results previously found in adults with CP living in LMICs^12,15^ and HICs.^28^ This difference may reflect differing perspectives between individuals with CP and their TD peers, as satisfaction is often shaped by personal expectations.

### Associations between fatigue and life habits

In the current study, no statistically significant associations were found between self-perceived fatigue and level of accomplishment or satisfaction of adolescents and adults with CP living in urban South Africa. This suggests that fatigue did not influence the participants’ daily activities and participation. This finding contrasts with studies conducted in HICs, where fatigue has been reported to impact daily participation^29^ as well as influence level of satisfaction.^7^ Differences in availability of resources between HICs and LMICs may have caused differences in the perception of self-perceived fatigue, which could in turn have an effect on a possible association with level of participation. For example, individuals with CP in a LMIC may walk long distances despite their physical limitations,^8^ and therefore may not perceive themselves as fatigued after covering certain distances. In contrast, individuals in a HIC, who may be less accustomed to walking such distances, could report higher fatigue scores for the same amount of walking.

### Limitations

A convenience, relatively small, sample of adolescents and adults with CP living in an urban area of Cape Town, South Africa, were recruited to participate in this study. Therefore, findings from this study cannot be generalized for all individuals with CP living in LMICs. In addition, this study used a cross-sectional design with two independent cohorts of adolescents and adults with CP. Future research should include a longitudinal study that follows up with adolescents with CP who grow into adulthood in order to identify the challenges encountered throughout the transition phase. In addition, it is imperative to understand that this study was focused on a relative brief (nine-item) and unidimensional questionnaire, based on the self-perceived effect of fatigue on daily activities of individuals living in a LMIC.

## Conclusions

Adolescents and adults with CP did not report more self-perceived fatigue compared to TD peers, and the majority of these individuals were independent and satisfied with their abilities to perform their daily activities and participate in the community. Furthermore, self-perceived fatigue was not related to accomplishment and satisfaction levels in individuals with CP living in a LMIC like South Africa. However, differences were observed in the levels of accomplishment and satisfaction with life habits between the adolescents and adults with CP compared to their peers.

Self-perceived fatigue in individuals with CP living in urban South Africa was relatively low compared to individuals living in HICs. This is presumed to be due to differences in experiences related to privileges.^10^ Besides the effect on the individual's mental health (e.g., resilience, coping strategy), limited access to transport might urge individuals with CP living in LMIC to be more active, resulting in better fitness levels. For future research, it would be interesting to objectively measure fatigue (using the International Classification of Functioning, Disability and Health model of body structure and function level),^5^ and determine if there is a relationship with the level of physical activities and fitness of this cohort living in a LMIC. This might provide insight to promote the deconditioning cycle as has been noted in individuals with CP ageing in HICs.^3^

## Appendix A Life-H scores for 12 dimensions and total weighted scores for level of accomplishment for adolescents and adults living with CP and TD peers.

**Table:**

	Adolescents		Adults	
	CP (n = 31)	TD (n = 31)	CP (n = 30)	TD (n = 30)
	Median [IQR]	Median [IQR]	Median [IQR]	Median [IQR]
Nutrition	7.8 [5.6–7.8]	7.8 [7.8–7.8]	9.3 [6.1–10.0]	10.0 [10.0–10.0]
Fitness	9.3 [8.5–10.0]	10.0 [10.0–10.0]	9.4 [7.3–10.0]	10.0 [9.4–100]
Personal care	7.9 [7.5–9.4]	10.0 [10.0–10.0]	9.6 [6.7–10.0]	10.0 [10.0–10.0]
Communication	9.7 [9.4–10.0]	10.0 [10.0–10.0]	10.0 [10.0–10.0]	10.0 [10.0–10.0]
Housing	6.2 [3.7–10.0]	10.0 [6.2–10.0]	7.7 [5.3–9.3]	10.0 [8.5–10.0]
Mobility	6.0 [3.0–10.0]	10.0 [10.0–10.0]	6.8 [4.8–8.6]	10.0 [10.0–10.0]
Responsibilities	5.6 [4.1–9.4]	10.0 [7.8–10.0]	10.0 [8.4– 10.0]	10.0 [10.0–10.0]
Interpersonal relationships	10.0 [10.0–10.0]	10.0 [10.0–10.0]	10.0 [9.7–10.0]	10.0 [10.0–10.0]
Community life	7.8 [3.7–10.0]	10.0 [10.0–10.0]	8.5 [5.4–10.0]	10.0 [10.0–10.0]
Education	10.0 [10.0–10.0]	10.0 [10.0–10.0]	10.0 [10.0–10.0]	10.0 [10.0–10.0]
Employment	8.9 [4.4–10.0]	10.0 [10.0–10.0]	7.7 [3.9–9.8]	10.0 [10.0–10.0]
Recreation	6.2 [3.3–10.0]	10.0 [10.0–10.0]	7.1 [2.2–9.0]	10.0 [10.0–10.0]
Weighted score				
Level of Accomplishment	8.3 [6.0–9.4]	9.5 [9.4–9.6]	8.4 [7.0–9.2]	9.9 [9.6–10.0]

Abbreviations: Life-H, Life-Habits Questionnaire; CP, cerebral palsy; TD, typically developing; and IQR, interquartile range.

## Appendix B. Life-H scores for 12 dimensions and total weighted scores for level of satisfaction for adolescents and adults living with CP and TD peers.

**Table:**

	Adolescents		Adults	
	CP (n = 31)	TD (n = 31)	CP (n = 30)	TD (n = 30)
	Median [IQR]	Median [IQR]	Median [IQR]	Median [IQR]
Nutrition	10.0 [8.8–10.0]	10.0 [6.3–10.0]	5.7 [5.0–10.0]	10.0 [8.5–10.0]
Fitness	8.3 [5.0–10.0]	10.0 [6.7–10.0]	5.0 [2.5–10.0]	10.0 [5.0–100]
Personal care	5.7 [5.0–10.0]	7.1 [5.8–10.0]	5.0 [5.0–10.0]	10.0 [7.2–10.0]
Communication	9.4 [5.0–10.0]	10.0 [6.3–10.0]	10.0 [5.0–10.0]	10.0 [8.3–10.0]
Housing	5.0 [5.0–5.0]	5.0 [5.0–7.1]	5.0 [4.9–7.7]	9.4 [5.0–10.0]
Mobility	5.0 [3.3–5.0]	6.7 [5.0–10.0]	5.0 [1.7–6.4]	10.0 [5.0–10.0]
Responsibilities	5.0 [5.0–10.0]	10.0 [5.0–10.0]	7.5 [5.0–10.0]	10.0 [7.5–10.0]
Interpersonal relationships	10.0 [7.5–100]	9.0 [5.8–100]	7.5 [5.0–10.0]	10.0 [5.5–10.0]
Community life	5.0 [2.5–7.5]	10.0 [5.0–10.0]	5.0 [4.2–9.4]	8.1 [5.0–10.0]
Education	5.0 [5.0–10.0]	10.0 [5.0–10.0]	7.5 [5.0–10.0]	10.0 [5.0–10.0]
Employment	6.0 [5.0–10.0]	10.0 [8.8–10.0]	5.0 [0.0–7.3]	10.0 [5.0–10.0]
Recreation	5.0 [2.5–10.0]	10.0 [5.0–10.0]	6.0 [2.8–10.0]	10.0 [6.7–10.0]
Weighted score				
Level of Satisfaction	6.5 [5.2–8.8]	9.0 [6.1–9.2]	5.6 [5.0 −7.8]	9.1 [6.6–9.7]

Abbreviations: Life-H, Life-Habits Questionnaire; CP, cerebral palsy; TD, typically developing; and IQR, interquartile range.
